# Supplementary figures and images for: CRISPR loss of function screening to identify genes involved in human primordial germ cell-like cell development
Source: PLoS Genet. 2023 Dec 13;19(12):e1011080. doi: 10.1371/journal.pgen.1011080 (PMC10752514; doi:10.1371/journal.pgen.1011080)

# S1 Fig

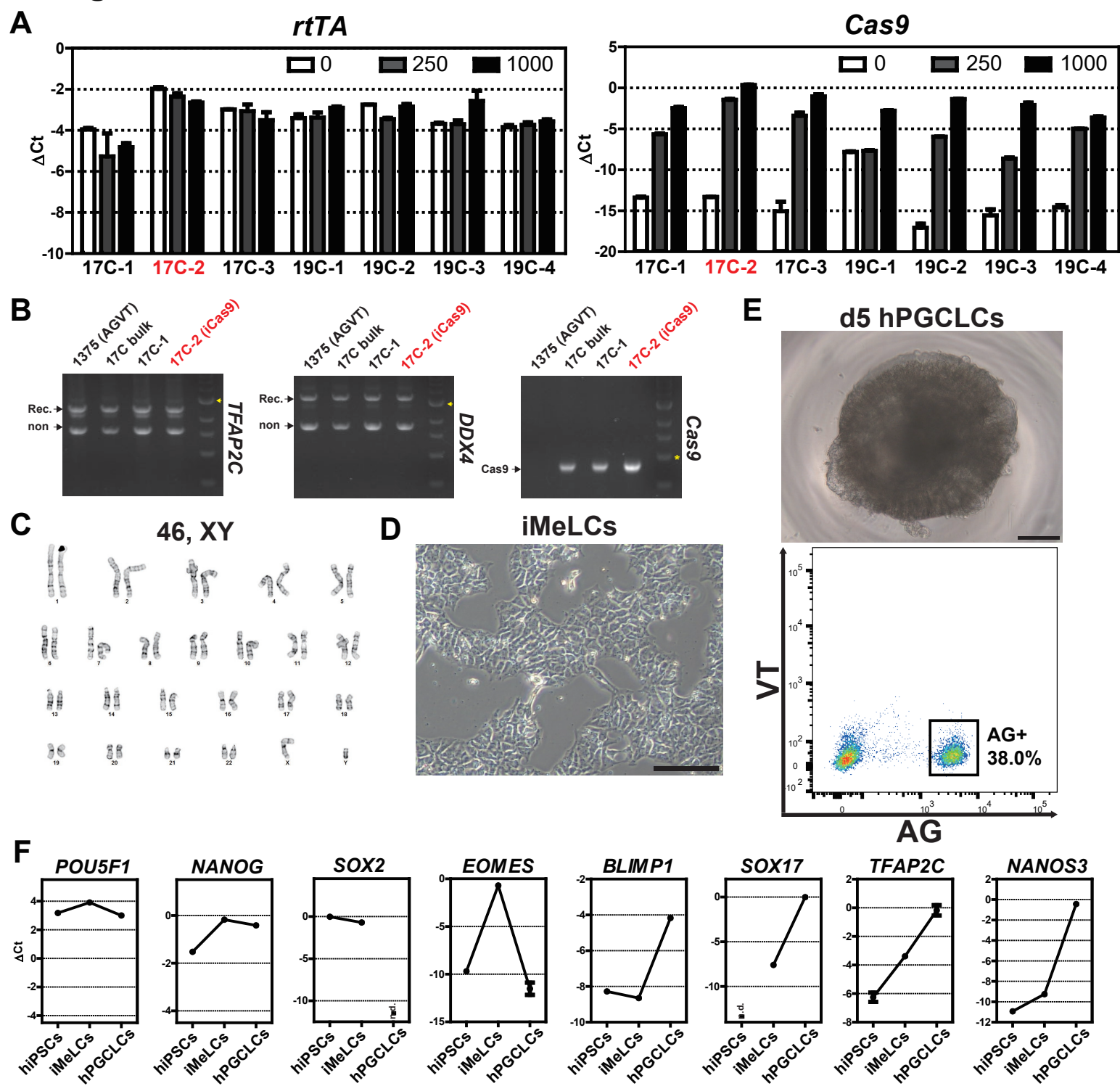

Supplement: S1 Fig — (A) Gene expression of rtTA (left) and Cas9 (right) in the indicated hiPSC clones. White, gray and black bars indicate cells treated with Dox at 0, 250 or 1000 ng/ml, respectively. The quantification of the gene expression levels was as shown in Fig 1E. Error bars indicate SD of technical duplicates. Clone 17C-2 (highlighted in red) was selected for downstream CRISPR screening assays because it showed the highest Cas9 expression after Dox treatment. (B) PCR genotyping of the TFAP2C-2A-EGFP (AG) (left), DDX4-2A-tdTomato (VT) (middle) and Cas9 coding sequence (right). Rec., recombined with fluorescent protein; non, non-targeted; yellow arrow, 3 kb; yellow asterisk, 1 kb. (C) Representative results of 17C-2 hiPSC karyotype analysis, showing a normal karyotype (46, XY). (D) Phase-contrast image of 17C-2 hiPSC-derived iMeLCs. Bar, 200 μm. (E) Bright-field image of a day 5 floating aggregate derived from 17C-2 hiPSCs (top) and its FACS plot. Bar, 200 μm. The percentages of AG+ cells (highlighted in box) are shown. (F) Gene expression dynamics of key markers during hPGCLC induction from iCas9 hiPSCs. hPGCLCs were harvested on day 5. Error bars indicate the standard deviation (SD) of technical duplicates. n.d., not detected. (PDF) [file pgen.1011080.s001.pdf]

**S2 Fig**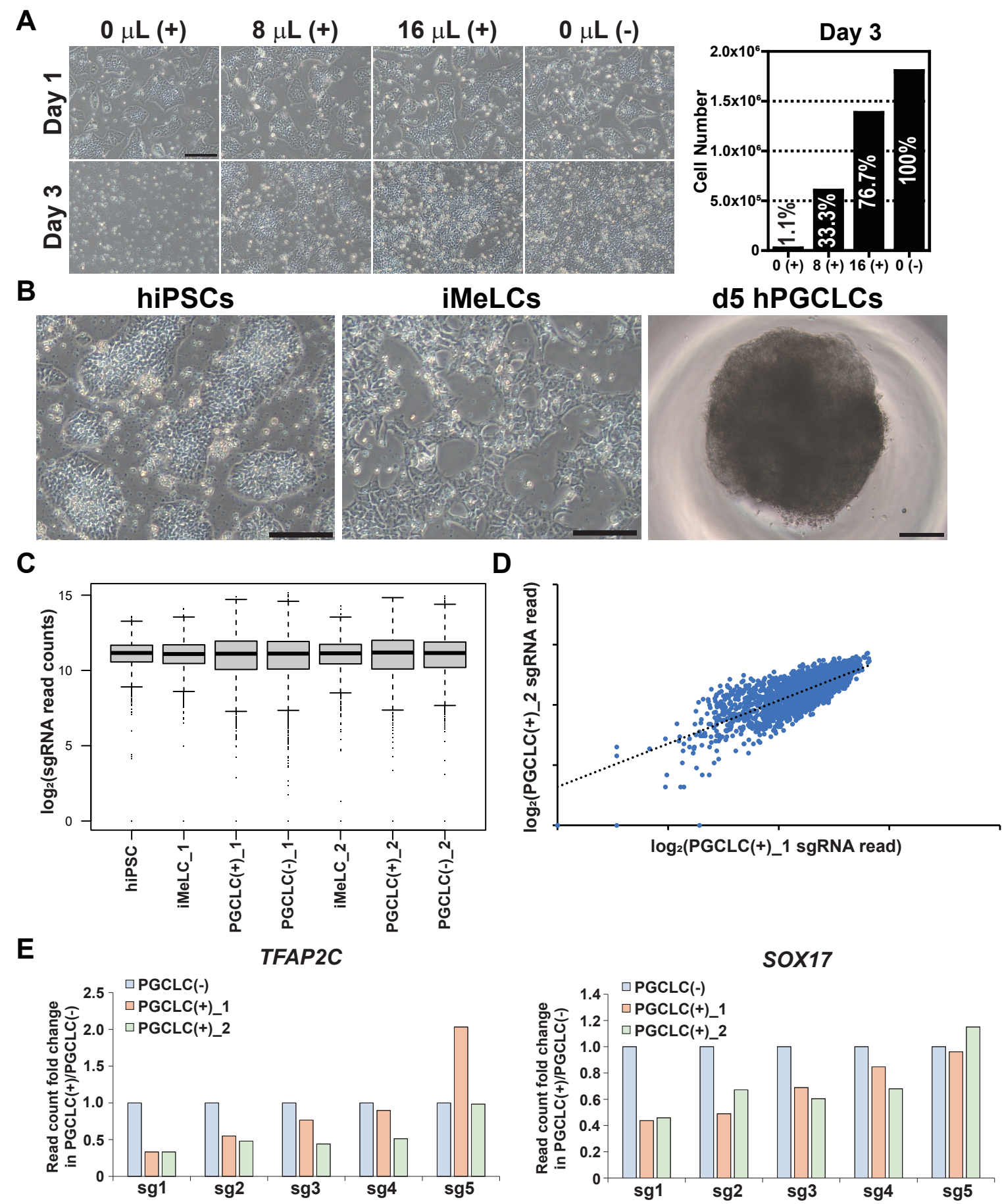

Supplement: S2 Fig — (A) (left) Phase-contrast images of lenti-viral transduced 17C-2 hiPSCs before (day 1) and after (day 3) puromycin selection. Volumes of virus-containing supernatant added per well (0 μl, 8 μl and 16 μl; virus titer) are indicated. (+), with puromycin; (-), without puromycin. (right) The number of cells counted at day 3 in each titer. 0 (-) is set as 100%. Titer 8 was selected as the optimized titer for a multiplicity of infection (MOI) of 0.3. (B) Phase-contrast images of 17C-2-CC hiPSCs (left), iMeLCs (middle) or day 5 floating aggregates containing hPGCLCs derived from 17C-2-CC hiPSCs (right). Bars, 200 μm. (C) Distribution of abundances of normalized sgRNA read counts in all screen samples. (D) Comparison of read counts of all sgRNAs in biological replicates of PGCLC(+) screen samples. (E) Normalized read counts of sgRNAs targeting TFAP2C (left) and SOX17 (right) in PGCLC(+) vs. PGCLC(-) populations in screen replicates. Read counts in in PGCLC(-) cells set to 1.0. (PDF) [file pgen.1011080.s002.pdf]

**S3 Fig****A**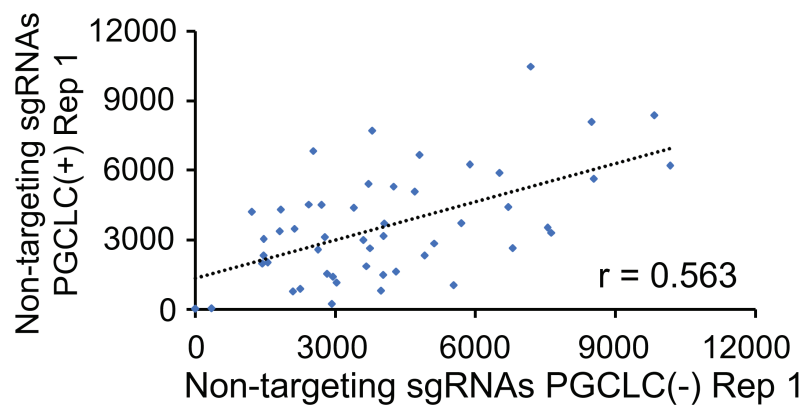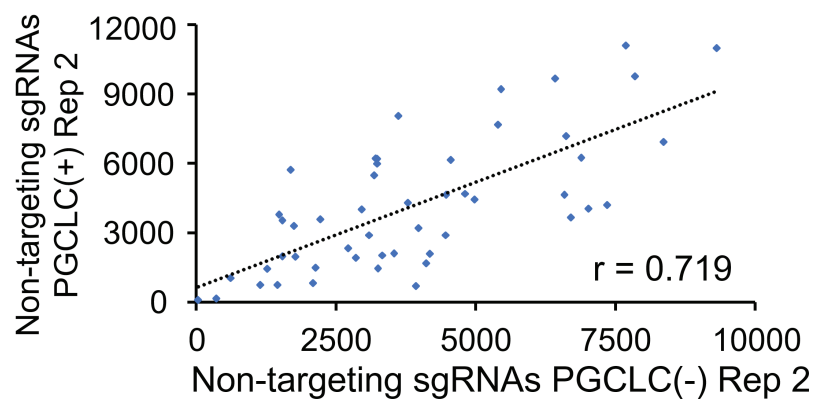**B**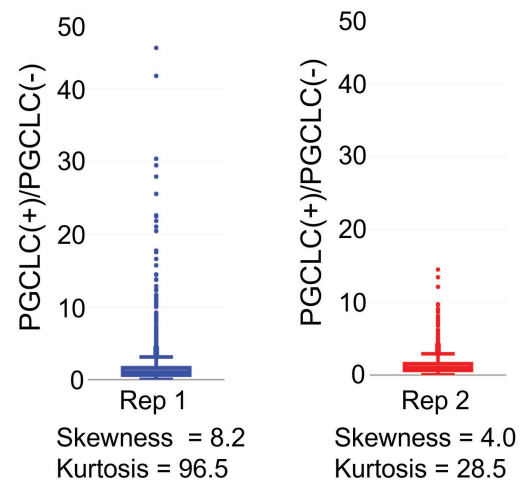**C**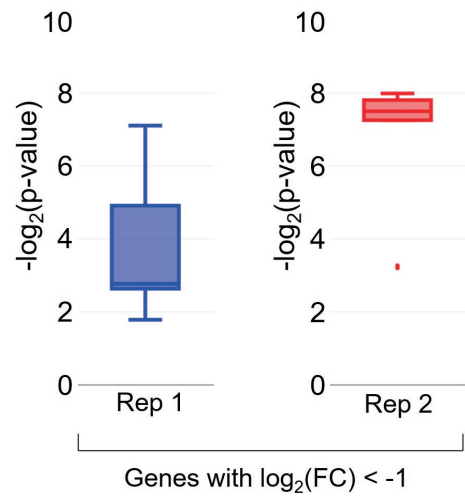

Supplement: S3 Fig — (A) Scatterplots showing correlation between non-targeting sgRNAs in hPGCLC(+) vs. hPGCLC(-) in screen replicates 1 (top) and 2 (bottom). r = Pearson correlation coefficient. (B) Distribution of fold changes in hPGCLC(+)/hPGCLC(-) cells in replicates 1 (left) and 2 (right). Deviation from normal distribution quantified by skewness and kurtosis. (C) -log2 p-values of genes depleted more than two-fold in hPGCLC(+) vs. hPGCLC(-) in replicates 1 (left) and 2 (right). (PDF) [file pgen.1011080.s003.pdf]

# S4 Fig

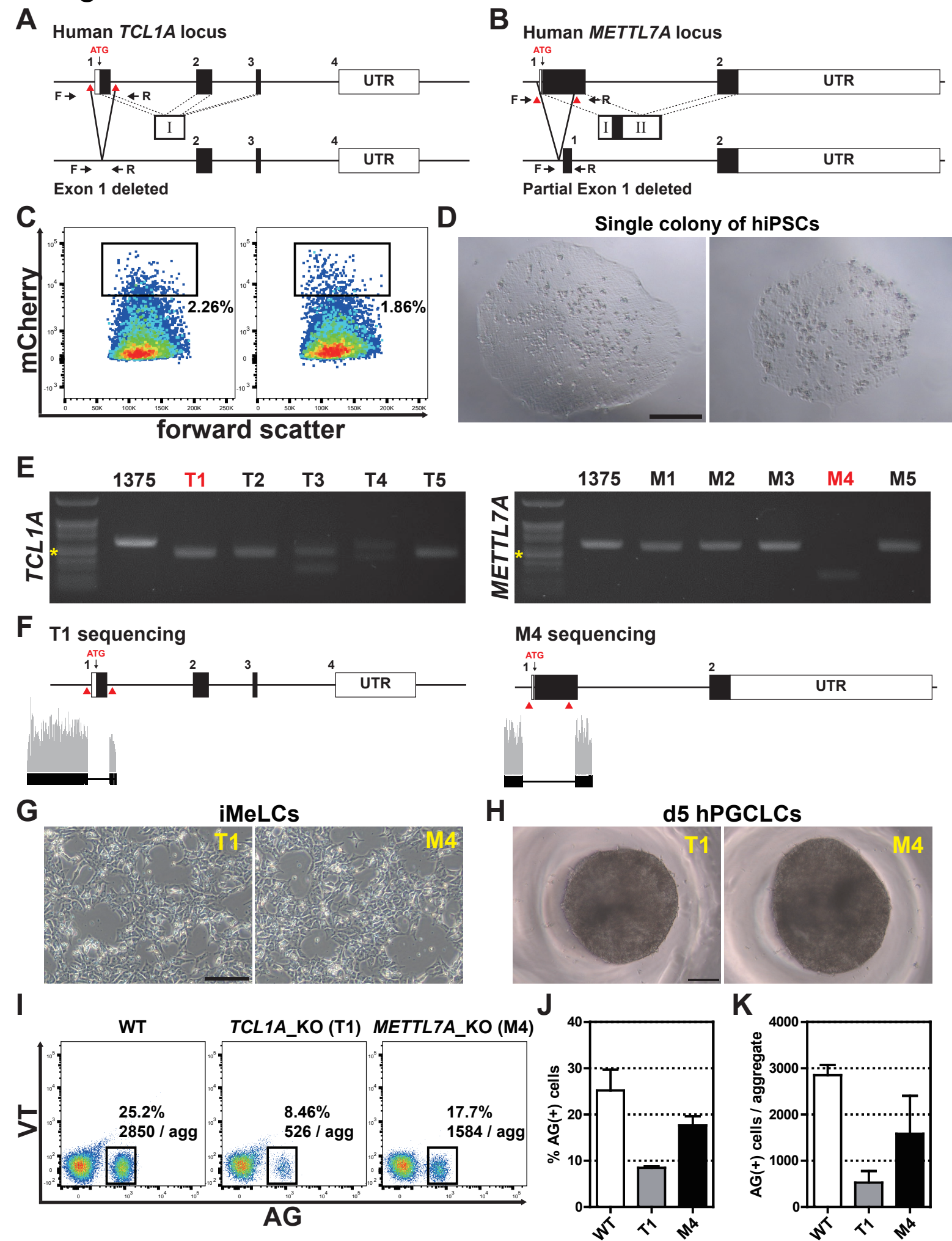

Supplement: S4 Fig — (A, B) Knockout schemes for human TCL1A (A) or METTL7A (B) loci. I in A, TCL1_MTCP1 domain; I and II in B, METTL7A signal peptide and Methyltransf_11 domain, respectively. Black boxes indicate exons. Red arrowheads indicate target sites recognized by the pair of Cas9 nickases flanking the start codons and the domain-encoding regions. Arrows indicate primer sites for genotyping. (C) FACS sorting of 1375 hiPSCs (WT) on the basis of mCherry expression by px335-derived nickase vectors targeting TCL1A (left) and METTL7A (right). The percentage of mCherryhigh cells (highlighted in boxes) and the sorting gates are shown. (D) Phase-contrast images of single clonal expansion of TCL1A- and METTL7A-targeted hiPSCs. Bar, 200 μm. (E) PCR genotyping of the large deletion in TCL1A (left) and METTL7A (right) exons by double pairs of nickase. Yellow asterisk, 500 bp. T1 and M4 (red) clones were selected for further analysis of each knockout line. (F) Sanger sequencing results of large deletions in T1 (left) and M4 (right). The thin black line represents a gap between target sites recognized by the pair of Cas9 nickases. (G, H) Phase-contrast images of iMeLCs (G) and day 5 floating aggregates containing hPGCLCs (H) derived from T1 (left) and M4 (right). Bars, 200 μm. (I) FACS analysis of day 5 hPGCLCs derived from WT (left), T1 (middle) and M4 (right). Boxes indicate AG+ cells. The average percentage and number of AG+ cells per aggregate from two independent experiments are also shown. (J, K) Percentage of AG+ cells (J) and number of AG+ cells per aggregate (K) in WT (white), T1 (gray) and M4 (black) in day 5 hPGCLCs. Error bars indicate SD of biological replicates. (PDF) [file pgen.1011080.s004.pdf]

**S5 Fig**

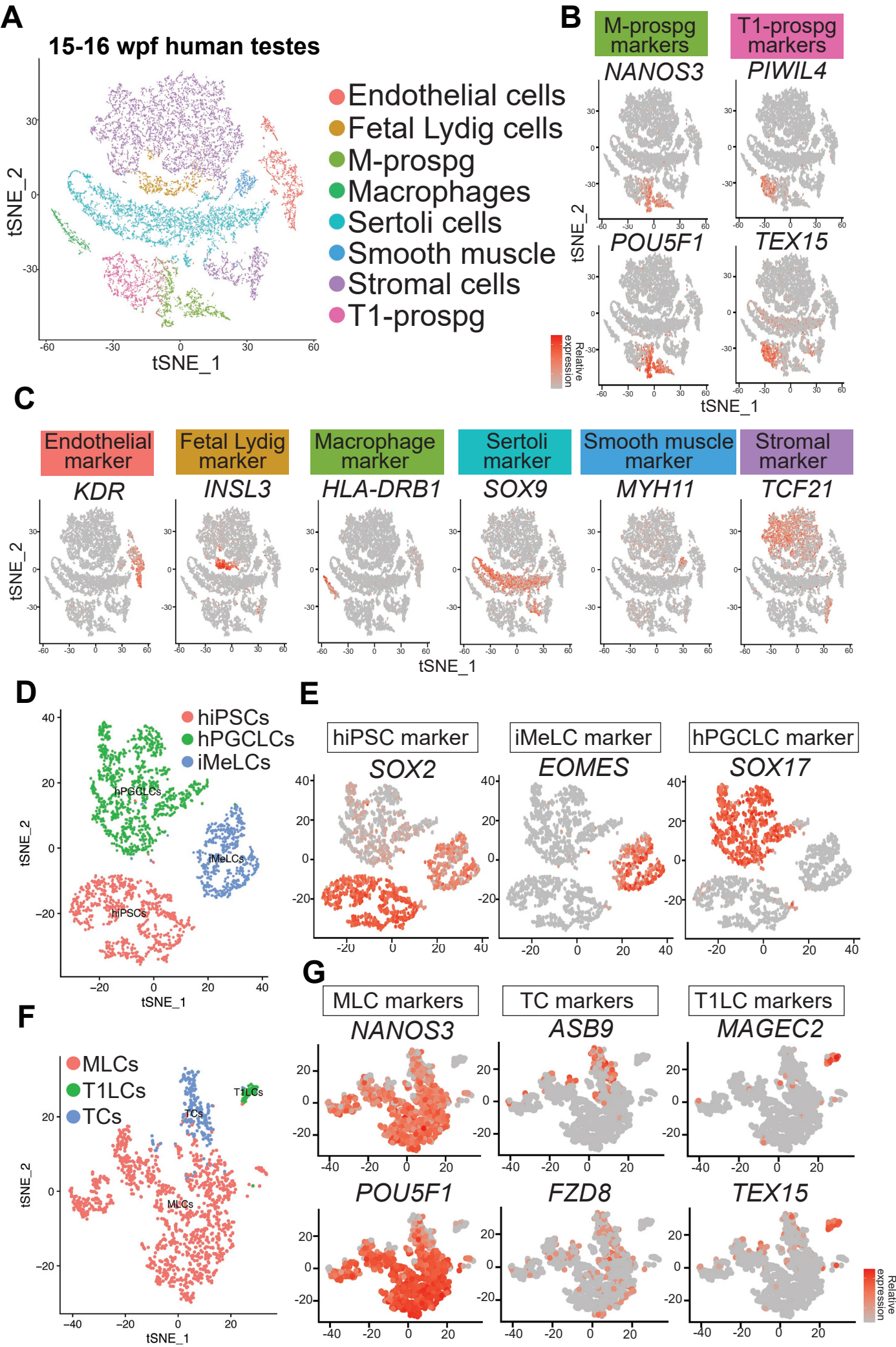

Supplement: S5 Fig — (A) tSNE plot showing different cell types in human fetal testes at 15–16 week post fertilization (wpf) [9]. Cell types were assigned based on the known markers as shown in B and C and colored accordingly. (B, C) Expression of key marker genes associated with indicated cell types [9]. (D, E) scRNA-seq data of hiPSC-derived cells during hPGCLC induction originated from three samples (hiPSCs, iMeLCs, hPGCLCs) were projected on tSNE and annotated based on the key markers as shown in (E). (F, G) scRNA-seq data of hiPSC-derived germ cells obtained from xenogeneic reconstituted testes (xrTestes) were annotated based on key markers as shown in (G). (PDF) [file pgen.1011080.s005.pdf]

# S6 Fig

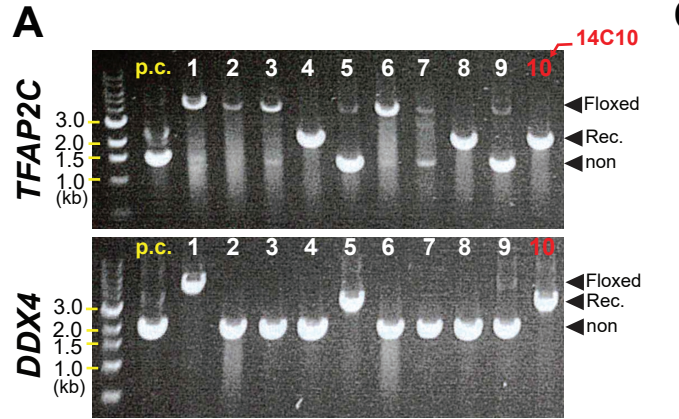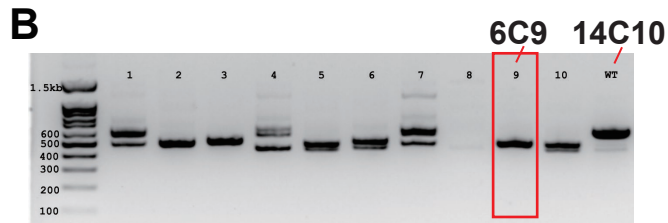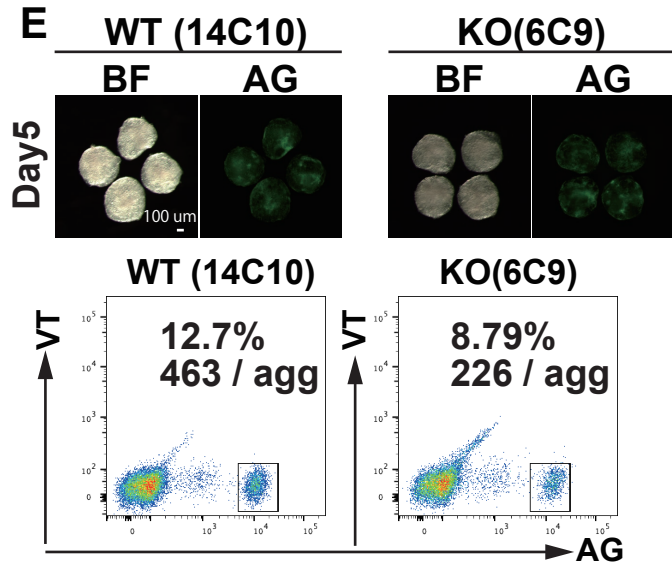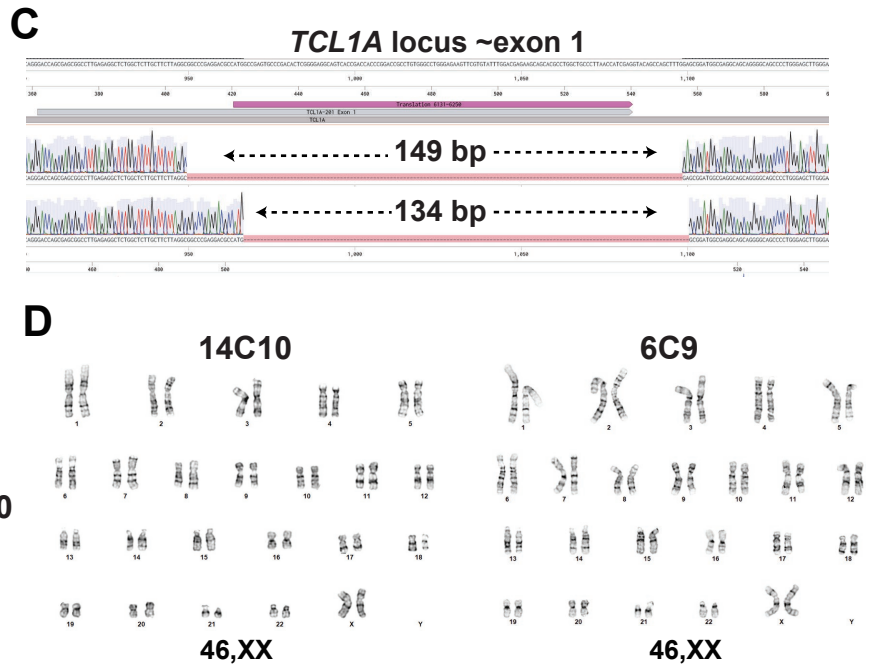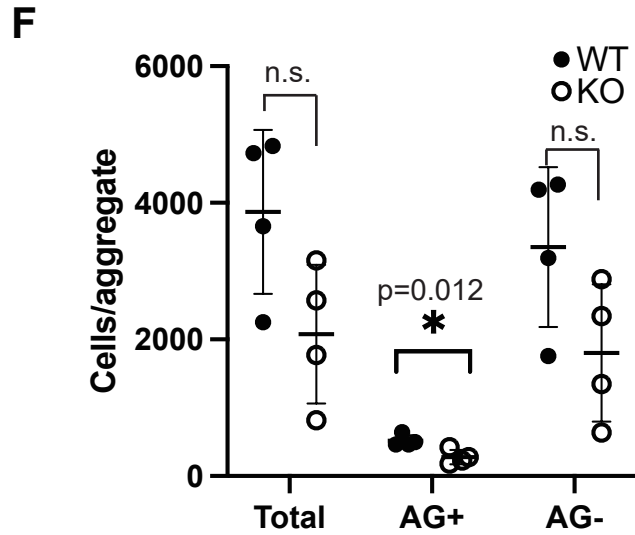

Supplement: S6 Fig — (A) Genotyping PCR to screen for clones bearing TFAP2C-p2A-EGFP (AG) (top) and DDX4-p2A-tdTomato (VT) alleles (bottom). Note that clone 10 (designated as 14C10 hiPSCs) bear biallelic AG and VT alleles. 585B1 1375 hiPSCs (monoalleleic for both AG and VT alleles) were used as positive control. (B) Genotyping PCR to screen for TCL1A mutant clones. Clone 9 (designated as 6C9 hiPSCs) was used for downstream assays. 14C10 hiPSCs were included as positive control. (C) Sequencing chromatogram of 6C9 hiPSCs at TCL1A loci showing bialleleic deletion (134 and 149 bp). (D) Representative results of karyotype analysis for 14C10 and 6C9 showing normal female karyotypes. (E) (top) Bright field (BF) and fluorescence (AG) images of floating aggregates at day 5 after hPGCLC induction from wild-type (WT, 14C10, left) or TCL1A KO (KO, 6C9, right) hiPSCs. (bottom) FACS analysis of day 5 hPGCLCs (WT, left; KO, right) for AGVT expression. (F) The number of total cells, or AG+ or AG− cells in hPGCLC aggregates at day5. Statistically significant differences between WT (black circles) and KO (white circles) were identified with a two-tailed t-test. Means ± standard deviation are shown. n.s., not significant. (PDF) [file pgen.1011080.s006.pdf]
