# Supplementary material for: CRISPR loss of function screening to identify genes involved in human primordial germ cell-like cell development
Source: PLoS Genet. 2023 Dec 13;19(12):e1011080. doi: 10.1371/journal.pgen.1011080 (PMC10752514; doi:10.1371/journal.pgen.1011080)
Supplement: Attachment — Submitted filename: 127349_1_rebuttal_2768721_rzprmk.docx [file pgen.1011080.s014.docx]

Rebuttal: RC-2022-01500

We would like to sincerely thank the reviewers for their constructive comments, which we have used as the basis for revising our manuscript.

We have addressed the reviewers’ specific comments below.

**Reviewer’s comments:**
Reviewer #1 (Evidence, reproducibility and clarity (Required)):

*1. A major concern in this manuscript is whether TCL1A function is specifically involved in hPGC development or generally important for other cell type. As AKT signaling plays multiple roles on many cell contexts, this is important to verify the author's conclusion. For example, is there any defect in proliferation of TCL1A-knockout iPS lines? The authors should quantify the doubling rate of TCL1A-knockout iPS cells, the number of iMeLCs yielded, and the cell number included in the aggregates. Looking at Figure S3K and K, the total number of the cells in the aggregates seems lower in TCL1A-knockout aggregates than in WT.*

**Response 1.** By analyzing our scRNA-seq data and qPCR, we confirmed that TCL1A expression is uniquely seen in early germline development in vivo and in vitro and not seen in hiPSCs nor iMeLCs highlighting its role in early germline development (Fig. 3A-B in the revised manuscript). Moreover, we confirmed that there is no defective proliferation in either mutant hiPSCs or iMeLCs (Fig. 3D). As correctly pointed out by the reviewer, AG^+^, AG^−^ and total number of cells in aggregates were all affected in *TCL1A* KO aggregates compared to WT. This trend was confirmed by another *TCL1A* KO line we generated from different parental hiPSCs, which underscored the reproducibility of the finding. It is not clear how AG^−^ fraction has also been affected by *TCL1A* KO, but it might be due to 1) the functional role of *TCL1A* in AG^−^ cells, which include precursors of hPGCLCs, and cells at early mesodermal/endodermal lineage [1,2], 2) some AG^+^ cells have lost AG expression as reported previously [3], or 3) indirect influence of AG^+^ hPGCLCs on AG^−^ cells. Future studies will address these issues. These points were addressed in the revised manuscript (page 15, first paragraph).

*2. Related to the comment above, the author should add a statement describing expression pattern and level of TCL1A and METTL7A in tissue. Are they preferentially expressed in the germ line, or generally expressed in a broad range of tissue?*

**Response 2.** Our scRNA-seq analysis of in human fetal testes revealed that *TCL1A* expression is uniquely seen in M-prospermatogonia but not in any of the somatic cells. In vitro, TCL1A expression was also limited to early phase of germline development (hPGCLCs, M-prospermatogonia-like cells, transitional cells). Please also refer to Response 1.

*3. The quantification of pAKT and p-mTOR is vague. The authors should quantify in a different way. Although the author claimed that Western blot analysis was not able to detect pAKT and p-mTOR in PGCLCs, there are a number of reports that detect these proteins. As an advantage of PGCLC system is to handle a large number of cells in culture, the authors should perform rigorously the quantification.*

**Response 3.** We thank the reviewer for the comment. Western blotting requires a large number of cells, particularly when different proteins are analyzed (~5x10^5^ cells/assay at least in our hands), which is not practical even by using hPGCLCs. Such effort may not guarantee the more quantitative data than those provided by IF, either (western blot is typically considered to be “semi-quantitative”) [4]. In this study, we performed statistical analysis and confirmed the significant differences of the mean intensity as well as positive cell numbers. We also performed the functional assay (OP-puro incorporation assay). Therefore, constellation of findings support our conclusion.

*4. In the abstract, there is a statement "demonstrate the importance of translational control in human reproduction". Isn't this too general? Is it a new finding? With critical examination for cell-specificity of the translational control by TCL1A as described above, this should be refined.*

**Response 4.** This has been corrected to be more specific.

Reviewer #1 (Significance (Required)):

As authors performed CRISPR/Cas9-based screening to identify genes involved in human germ cell development using human PGCLC system, and then successfully isolated TCL1A and METTL7A genes that are previously known as drives for PGCLC induction. Therefore, from both technological and scientific viewpoints, there is a significant advance shown in this manuscript.

**Response 5.** We appreciate the positive comment from the reviewer.

Reviewer #2 (Evidence, reproducibility and clarity (Required)):

*Unfortunately, we found that the evidences for the key conclusions are not quite convincing. Reasons are as below:
1. The results to demonstrate the key role of TCL1A on hPGCLC specification is not convincing.
Fig. 3B, C & D. the cell number per aggregate is also significantly reduced in TCL1A KO (2843/20.7% =13734) compared to that in WT cells (545/16.4%=3323). Despite that, the percentage of AG+ cells per aggregate is significantly, but not dramatically decreased in TCL1A KO (20.7%) vs WT (16.4%) cells. Thus, the effect of TCL1A KO may not be specific on the AG+ cells, but on the whole aggregate.*

**Response 6.** We confirmed the finding by using an independent pair of wild-type and TCL1A knock-out lines. Please also see **Response 1**.

*2. The overall effect of METTL7A KO on hPGCLC development is too moderate to conclude it as a key regulator for hPGCLC specification.*

**Response 7.** We agree with the reviewer. Therefore, we have already toned down the role of METTL7A in hPGCLC specification (page 10, “TCL1A, and to a more moderate extent METTL7A, significantly impairs….”).

Reviewer #3 (Evidence, reproducibility and clarity (Required)):

*Specific Comments:

1.) p.7-p.8: "Using the MAGeCK algorithm (Li et al, 2014) to call hits on merged replicates, 25 genes scored as significantly depleted from the AG+ population at p < 0.05. Among the top hits was SOX17, and near-hits included TFAP2C, both of which are well-known drivers of the hPGCLC state (Fig. S2E)."

When looking at Table S3, it appears that only 2 genes were significantly depleted (P < 0.05) in replicate 1, 23 in replicate 2 and 10 genes when rep 1 and rep 2 were analyzed together. The essential germ cell genes SOX17 and TFAP2C were not significantly depleted in replicate 1 and only TFAP2C but not SOX17 was depleted significantly in replicate 2. Also the main hits discussed in this paper, METTL7 and TCL1A were not significantly depleted in replicate 1 and only METTL7 but not TCL1A was significantly depleted in replicate 2. This indicates that replicate 1 might not have been robust enough to reliably detect depleted genes and that TCL1A was not among the significant hits. A potential explanation could be that not enough cells have been used to ensure a sufficient representation of sgRNAs to provide significant results in a depletion screen. Ideally the screen would need to be repeated to provide another informative replicate, or the authors should at least correct the sentences above and openly state that their hits are only based on one replicate of the screen and that the list of their hits might therefore not be fully reliable. Also the statement on page 8 that "the screen was both technically and biologically successful" might need to be toned down.*

**Response 8.** We greatly appreciate the Reviewer's astute observations and critical feedback about our screens. First, we must clarify the issue of the statistical significance of the screen hits TCL1A and METTL7A. There are multiple ways of utilizing the MAGeCK algorithm to analyze screening data, and we had intended to show results from multiple implementations to be thorough. Due to an oversight in preparing the Supplementary Tables, for each screen replicate analysis we only included tables showing one such implementation in which we compared the PGCLC(+) vs. *only* the sorted PCGLC(-) population. This analysis has less statistical power than when we compare PGCLC(+) to *both* PGCLC(-) and iMELCs (as iMELCs are also PGCLC(-)). In this latter, higher-powered analysis, in screen replicate #2 there are 30 genes scoring at p < 0.05. TCL1A has a p-value of 0.0056 and has the 5^th^ greatest log fold change of all hits, and METTL7A has a p-value of 0.0045 and has the second greatest log fold change of all hits. Importantly, the addition of the iMELC population does not change screen results qualitatively, only quantitatively (due to the increased statistical power obtained by adding another sample without increasing overall variance). Of the 23 hits scoring at p < 0.05 in the first analysis (without iMELC sample) of screen #2, 17 also score in the second analysis when the iMELC sample is added. Further, the correlation between log fold-changes of all genes in the two analysis is very high: r = 0.83 (Figure 1 of this document, below). Thus, both genes studied do in fact score highly significantly in screen replicate #2 when the analysis is improved by adding statistical power.

Also of importance is that adding in the iMELC sample does *not* change the overall results in screen replicate #1. TCL1A and METTL7A still have trends toward depletion that fall short of statistical significance. The reason is that, as pointed out by Reviewer 2, screen replicate #1 was not as effective overall, from a technical perspective, as replicate #2. We suspect that this was due to the lower coverage in this screen, which resulted in noisier data that makes it difficult to achieve high statistical significance. The fact that adding the iMELC sample to the PGCLC(-) sample did not help is further supports this point. The iMELC sample is expected to be similar, biologically, to the PGCLC(-) sample – which is why including it improved the quality of the screen replicate #2 analysis. But in replicate #1, the sgRNA composition in the iMELC sample was *not* highly similar to that of the PGCLC(-) sample – again, due to noisiness – and including it in the analysis thus increased the overall variance. Critically, however, sgRNAs targeting TCL1A and METTL7A in fact performed similarly in screen replicate #1 as they did in replicate #2. The magnitude of depletion of each sgRNA is very similar in both screens – this is shown in Figure 2F of the manuscript. The reason these similar sgRNA depletions failed to achieve statistical significance in replicate #1 is simply because the screen overall was noisy. Thus, for the same magnitude of change of sgRNAs as in replicate #2, there is less power to detect it as significant in replicate #1.

In summary, when the higher-powered analysis is used, TCL1A and METTLA score highly significantly in screen replicate #2. The log fold-changes of their sgRNAs were very similar in screen replicate 1, but neither gene achieved statistical significance in this case due to the higher screen noisiness. Our revised manuscript will include the high-powered screen analysis, and we greatly apologize for the oversight of not including it in the initial submission. Additionally, we will discuss the importance of achieving high screen coverage and will also adjust our language so as to more accurately reflect our screen results.

**Figure 1.** Correlation of log fold-changes for all genes in screen replicate #2 when the iMELC sample is (y-xis) or is not (x-axis) included with the PGCLC(-) sample in the MAGeCK analysis.

*2.) p.8 and Fig. 2E: The authors do not clearly describe, what are the 25 top hits in PGCLC(+) and PGCLC(-) cells and how they were chosen (Score, p-value or fold change), which they compared in the gene set enrichment analysis to the RNA-Seq data.*

**Response 9.** We apologize for not describing this clearly. These are the top and bottom 25 genes, ranked by p-value, in the comparison of the PGCLC(+) samples vs. the iMELC samples, using samples from both screen replicates. iMELC samples were used to represent PGCLC(-) cells in this case because the iMELC populations are fairly similar between the two screen replicates, while the sorted PGCLC(-) samples are rather dissimilar. "Top" 25 genes refers to those with sgRNAs most depleted from the PGCLC(+) population (ie. genes predicted to be required for progression to PGCLCs), and "Bottom" 25 genes refers to genes most enriched in the PGCLC(+) population (ie. genes predicted to be repressing the progression to PGCLCs). We have made this clearer in the revised manuscript.

*3.) Fig. S3I-K: The authors mention in the text on p. 9 a significant reduction in hPGCLC induction efficiency for both TCL1A and METTL7A KO cells, but they do not provide statistics and do not mention, how many biological replicates have been used. As hiPSCs generally show a high clone to clone variability in hPGCLC induction efficiency, results from a single KO clone can not be considered as a reliable result. The authors should provide results from additional wt and KO clones (they are showing in S3E multiple for each gene) to ensure reliable effects, especially for METTL7A KO cells, where the reduction in PGCLC induction efficiency is more modest and might not be significant (Fig. S3I). Another way of validating the phenotype would be to use individual control, TCL1A and METTL7A sgRNAS from the screen and compare induction efficiencies with or without DOX-induced Cas9 expression.*

**Response 10.** We thank the reviewer for pointing out the scientific rigor related to our experiments. We carefully addressed the effect of *TCL1A* on hPGCLC induction with increasing biological replicates now using another set of *TCL1A* KO and WT lines derived from independent hiPSC cells bearing fluorescence reporter alleles AGVT (14C10, please see METHODS). Please also see the comment above.

*4.) Fig. 3F, Supp Tables S4, S5, S6: It is not clearly described, what was the criteria to define DEGs for the GO term analysis of TCL1A KO cells, as more genes have been used than the relatively few significant DEGs reported in Table S4. Furthermore, only FDRs or otherwise adjusted p-values (not raw p-values as done in the figure and tables) should be used to determine significantly enriched GO terms. Also no representative gene names are displayed in Fig.3F, as stated in the figure legend.*

**Response 11.** This point has been addressed and new description has been added to the figure legend.

*5.) Fig.4: p-AKT and p-mTOR signaling are represented as the mechanism, by which TCLA1 KO affects hPGCLC maintenance/proliferation. It is not clear from the presented data, what is meant with biological triplicates (different germ cell inductions, different subclones) mentioned in the figure legend. As some of the effects observed are quite small (e.g. p-mTOR differences in 4E, cell cycle differences in 4J), biological replicates with a different KO clone should be performed (see point 3). Otherwise it is hard to judge how robust the data and conclusions derived are.*

**Response 12.** We apologize for the confusing description. Biological replicates mean the replicates from different hPGCLC induction from a same KO and WT pair. Please also refer to Response 3.

*CROSS-CONSULTATION COMMENTS
Apart from my points regarding the weakness in statistical confidence I agree with the other reviewers that it needs to be shown whether the effect of TCL1A KO is based on a general proliferation defect of the entire aggregation body or if the effect is really hPGCLC-specific.*

**Response 13.** Please refer to Response 1.

*Reviewer #3 (Significance (Required)):

The study is the first CRISPR screen performed during hPGCLC differentiation and provides a proof of principle for a useful tool to allow dissection of gene regulatory networks during human germ cell development. Overall this is a technical advancement and an ambitious study and will generate interest in the human germ cell field. Generally it is easy to follow but could be improved in the description of some of the methodology (points 2+4). Overall the study suffers from weaknesses in the statistical robustness, as the screen itself did not provide many significant hits (major point 1) and the follow up was only performed using a single KO clone (points 3+5). Therefore adding replicates would be necessary to strengthen the confidence in the drawn conclusions.
Reviewers' relevant expertise: in vitro germ cell differentiation, pluripotency, CRISPR screening*

**Response 14.** Regarding major point 1 (weaknesses in the statistical robustness of the screen analysis and low number of screen hits): please see our response to Reviewer #2. We believe that the higher-powered MAGeCK analysis we describe in that response addresses the statistical robustness of the screen analysis and provides an explanation for why screen replicate #2 yielded more hits than replicate #1, despite many similarities in the two screens.

**REFERENCES**

1.	Sasaki K, Yokobayashi S, Nakamura T, Okamoto I, Yabuta Y, Kurimoto K, et al. Robust In Vitro Induction of Human Germ Cell Fate from Pluripotent Stem Cells. Cell Stem Cell. 2015;17: 178–194. doi:10.1016/j.stem.2015.06.014

2.	Kojima Y, Sasaki K, Yokobayashi S, Sakai Y, Nakamura T, Yabuta Y, et al. Evolutionarily Distinctive Transcriptional and Signaling Programs Drive Human Germ Cell Lineage Specification from Pluripotent Stem Cells. Cell Stem Cell. 2017;21: 517-532.e5. doi:10.1016/j.stem.2017.09.005

3.	Murase Y, Yabuta Y, Ohta H, Yamashiro C, Nakamura T, Yamamoto T, et al. Long-term expansion with germline potential of human primordial germ cell-like cells in vitro. EMBO J. 2020;39: e104929. doi:10.15252/EMBJ.2020104929

4.	Taylor SC, Berkelman T, Yadav G, Hammond M. A defined methodology for reliable quantification of western blot data. Mol Biotechnol. 2013;55: 217–226. doi:10.1007/S12033-013-9672-6/FIGURES/7
