# Supplementary material for: CRISPR loss of function screening to identify genes involved in human primordial germ cell-like cell development
Source: PLoS Genet. 2023 Dec 13;19(12):e1011080. doi: 10.1371/journal.pgen.1011080 (PMC10752514; doi:10.1371/journal.pgen.1011080)
Supplement: Attachment — Submitted filename: reviewer_comments_2.docx [file pgen.1011080.s015.docx]

**Rebuttal: PGENETICS-D-23-01001**

We would like to sincerely thank the reviewers for their constructive comments, which we have used as the basis for revising our manuscript. We have addressed the reviewer’s specific comments below.

**Reviewer's comments:**

*1. TCL1A expression and function in non-hPGCLC cells in embryoid body
As the other reviewers pointed out, loss of TCL1A likely impacts non-hPGCLC cells in embryoid bodies. In the revision, the authors have added scRNA-seq data to claim the specific expression in germ cells during male germ cell development, but it does not address whether non-hPGCLC cells in the embryoid bodies express TCL1A or not. Thus, there remains a concern that TCL1A might not be specific to hPGCLCs but rather a downstream target of cytokines used in the induction. The authors should confirm the expression by using their own or public scRNA-seq dataset, or immunostaining. If the expression is not specific in hPGCLCs, they should discuss it as a general role in early embryo development.*

**Response 1.** We sincerely thank reviewer #1 for the careful and critical evaluation of the manuscript. We have FACS-sorted AG (-) cells and analyzed their expression for *TCL1A* on day 2, 4, 6, 8. However, expression is essentially below our detection threshold by qPCR (Ct value above 30), suggesting that AG (-) cells do not express detectable levels of *TCL1A* (please see below raw qPCR data). We have replaced Fig.3C with the new graph including both AG^+^ and AG^−^ cells and modified the main text accordingly: “.. TCL1A expression is limited to early stage germ cells [AG^+^ hPGCLCs, M-prospermatogonia-like cells…. (revised manuscript page 10, second paragraph).

*2. Lack of some key regulators for PGC development in the candidate genes
The authors claim that their CRISPR screening is working because hit candidates contain SOX17 and TFAP2C, both are critical transcriptional regulators for hPGCLC specification and development. So far, the author’s group and the others identified some other key regulators such as EOMES and PRDM1, which are included in a list of CRISPR screen targets, but they seem to be lower scores from the result of the screen. The authors should explain how it happened and discuss what are the technical limitations of their system.*
**Response 2.** This point has been explained in Discussion (page 16, third paragraph in the revised manuscript) as follows: “There are certain limitations to the approach taken. High content CRISPR screening has become a common, powerful forward genetics method that is highly effective in cell lines. However, CRISPR screening in primary cells with more heterogeneity, lower numbers of cells, and sensitive culturing conditions is more challenging. Further, cell fate-based screens, which often rely on surface markers and are sensitive to gating schemes, are more inherently challenging than proliferation/viability screens. Accordingly, it was not surprising that a degree of noise was observed in our screens and that some, but not all, positive control genes scored as hits. Ultimately, as with many screens performed in challenging contexts, it must be assumed that some hits will be false negatives. However, the detection of confirmation of true positive hits is evidence of screen success overall.”.

*3. Gene expression pattern of TCL1A knockout hPGCLCs
In the comparative analysis of hPGCLCs transcriptome between wildtype control and TCL1A knockout, the authors only show PCA and DEG analysis. To clarify TCL1A knockout does not affect key germ cell regulators, it would be better to show a representative gene expression dataset based on categories such as pluripotency, germ cells, and epigenetics. It is curious if the loss of TCL1A affects genes upregulated after specification such as KLF4 and TFCP2L1.*

**Response 3.** Table S4 have already clearly demonstrated that these key germ cell regulators are not among those differentially expressed. Nonetheless, gene expression data for representative germ cell specifier genes and pluripotency genes, *KLF4* and *TFCP2Ll* were added based on transcriptome data in the revised Fig. 3K.

*4. In Figure 4A and B, SOX17 alone is not sufficient to detect hPGCLCs.
Since SOX17 is also expressed in definitive endoderm, the use of SOX17 alone as a marker for hPGCLCs is not appropriate. A loss of TCL1A may also affect the proportion of cell types in embryoid bodies.*

**Response 4.** We have previously demonstrated that AG^−^ non-PGCLC population during induction do not express SOX17 (~0.03 copy/cell in non-hPGCLCs vs ~131 copy/cell in hPGCLCs based on qPCR) (Fig.2G in ref #5). Therefore, SOX17 alone serve as a reliable marker for detection of hPGCLCs in our platform regardless of proportion of cell types in non-hPGCLC fraction. Moreover, neither iMeLCs (where early mesodermal and endodermal genes are upregulated) nor hPGCLCs show alternation of endodermal gene expression, further suggesting that loss of *TCL1A* does not affect endodermal differentiation (Fig. R1, figure for reviewers).
